# Exploiting Plasmonic Phenomena in Polymer Optical Fibers to Realize a Force Sensor

**DOI:** 10.3390/s22062391

**Published:** 2022-03-20

**Authors:** Francesco Arcadio, Luigi Zeni, Nunzio Cennamo

**Affiliations:** Department of Engineering, University of Campania Luigi Vanvitelli, Via Roma 29, 81031 Aversa, Italy; francesco.arcadio@unicampania.it (F.A.); luigi.zeni@unicampania.it (L.Z.)

**Keywords:** surface plasmon resonance (SPR), force optical fiber sensors, plastic optical fibers (POFs), multimode POFs, plasmonic force sensors

## Abstract

In this work, a novel sensing approach to realize a force optical fiber sensor is designed, developed, and experimentally tested. The proposed sensing methodology exploits the effects of deformation due to an applied force on a patch of plastic optical fiber (POF) connected at the input of a surface plasmon resonance (SPR) sensor realized in a D-shaped POF. Therefore, the proposed force sensor system consists of an SPR D-shaped POF sensor, connected to a spectrometer, within input of a POF patch, connected to a light source used for interacting with the applied force. When the applied force on the patch changes, the mode profile of the light in the multimode POF patch and the SPR-POF sensor change too, so the SPR spectra shift. The obtained experimental results demonstrate that the proposed sensor has a resolution of the force sensor equal to about 22 mN and an excellent linear response in the range from 0 N to 0.5 N.

## 1. Introduction

Sensors based on optical fibers have recently been developed for several sensing applications to exploit many advantages in respect to other sensors. In fact, this kind of optical sensor presents attractive properties very useful to realize efficient sensor systems, such as small dimensions, remote sensing capabilities, low cost and light weight. So, to measure both physical quantities and specific substances, different optical fiber sensors and bio/chemical sensors have been proposed by several research groups [1,2]. More specifically, optical fiber-based sensors for physical quantities have been developed to measure strain, temperature, force, pressure, magnetic field, volume, acoustic wave, liquid level, etc. [2,3,4,5]. These optical fiber sensors are based on different sensing approaches, such as surface plasmon resonance (SPR), fiber Bragg gratings (FBG), Fabry–Perot, etc. Moreover, several of these sensing methods are used to realize bio/chemical sensors to detect specific substances, exploiting these transducers combined with specific receptors [1,6,7].

In particular, various force optical fiber sensors, simple to realize and use, have been developed exploiting the characteristics of plastic optical fibers (POFs) [8] and other physical characteristics [9,10]. For instance, Leal-Junior et al. proposed interesting force sensors based on optical fibers, such as those based on POFs and FBG sensors embedded into custom 3D-printed elements [11,12].

Plasmonic phenomena can be used to realize, via optical fibers, highly sensitive methods for detecting slight refractive index variations at the interface between a metal nanofilm and a dielectric medium [13,14,15,16]. Numerous sensor configurations that take advantage of the SPR phenomenon, both in silica fibers and POFs, have been reported to realize bio/chemical sensors, temperature sensors, magnetic field sensors, and many others [17,18,19,20,21,22,23].

SPR sensors can be realized by exploiting mono mode or multimode optical fibers. In particular, SPR phenomena can be obtained simply by exploiting multimode optical fibers, e.g., plastic optical fibers and hetero-core fibers. In plasmonic sensors based on multimode optical waveguides, the convolution of different resonance wavelengths is obtained for a specific resonance condition, defined by a given angle–wavelength couple. So, when the input light undergoes variations, the shape of the SPR spectrum changes.

Cennamo et al. used this peculiarity in [23] to realize a novel sensing approach that, by exploiting SPR-POF sensors, has been used to carry out attractive magnetic field sensors. More specifically, Cennamo et al. monitored the resonance wavelength shift to measure the SPR spectra’s shape variation due to the interaction of a magnetic field with a patch covered by ferrofluids [23]. 

Consequently, starting from [23], an applied force sensor was developed, realized, and tested. These force optical fiber sensors could be used in several biomedical applications, such as Zhou et al. in [24], where an optical fiber photoacoustic sensor was used for real-time heparin monitoring. 

In this work, we explore how a POF patch fixed in a 3D-printed holder can be used to realize a sensitive force sensor. In more detail, the applied force on this patch changes the SPR conditions in input at an SPR-POF sensor connected to it by shifting the resonance wavelength (at a constant solution in contact with the sensing region of the SPR-POF sensor). The applied force range used in the experimental measurements was obtained by changing the weights applied on the patch fixed between the clamps of the developed holder. 

This work aimed to demonstrate the effect of applied forces on a multimode POF in a considered range exploiting plasmonic phenomena in POFs. In fact, an SPR-POF sensor has been used in an improper way here. It does not measure the refractive index variation in contact with the plasmonic sensing surface, but it is used for monitoring the modal light perturbation that occurs in multimode optical fibers. So, the proposed sensing method represents a novel approach for the production of optical fiber sensors, with a sensing region remote from the transducer (the SPR sensor) and the equipment. Moreover, the price of the SPR-POF sensor is about USD 3, and its setup is simple. Furthermore, an LED and a photodetector could be used instead of the white light source and the spectrometer.

The proposed sensor’s performances were obtained experimentally and compared with other optical fiber sensors in this work.

## 2. Principle of Sensing and Experimental Setup

**Figure 1:**
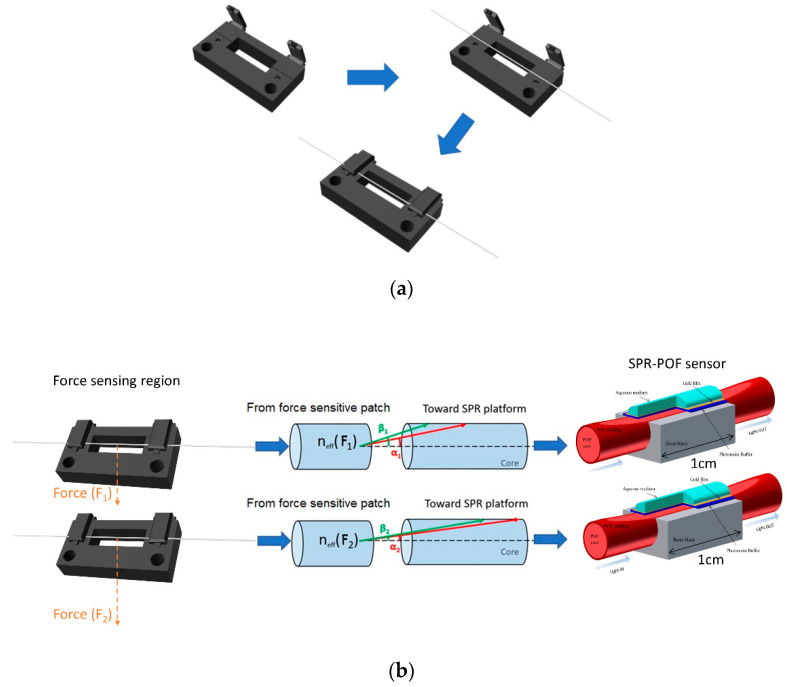

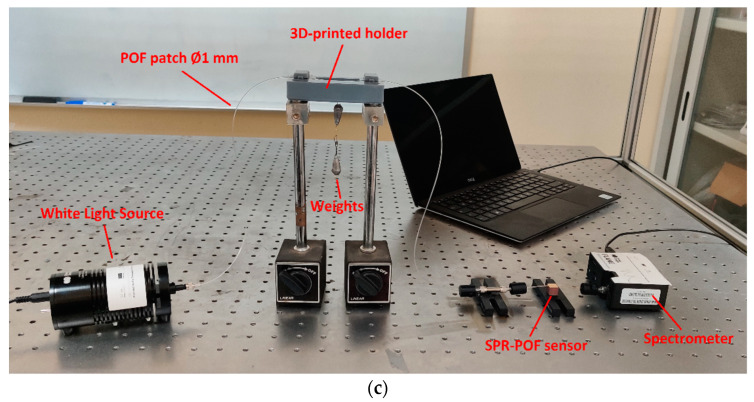
(**a**) 3D-printed holder to fix the patch of POF for the force interaction. (**b**) Outline of the proposed force sensing principle. (**c**) Picture of the experimental sensor configuration.

The proposed force sensor system consisted of a POF patch, fixed in a custom 3D-printed holder via two designed clamps to obtain the force sensing region, connected in input to an SPR D-shaped POF sensor. Regarding the connection at their other ends, the patch was connected with a light source, whereas the SPR-POF sensor was connected with a spectrometer. The developed 3D-printed holder used to realize the sensing region and apply the force on the POF is schematically shown in Figure 1a. This custom holder was designed and printed by a 3D printer (Photon Mono X UV Resin SLA 3D Printer, Anycubic^®^, Shenzhen, China). Figure 1a also shows the steps useful to fix the POF patch in the holder via clamps. 

To realize the patch and the SPR sensor, multimode step-index POFs were used. In detail, these POFs presented a total diameter of 1 mm (980 µm of core and 10 µm of cladding) with a core of PMMA and a fluorinated polymer cladding.

In the observation, the sensing principle consisted of the SPR wavelength shift at a constant refractive index of the bulk solution over the SPR-POF sensor, due to the changes of the exciting light ray angles caused by the applied force on the patch fixed between the clamps of the holder (see Figure 1b). In fact, by varying the applied force on the patch, the guided mode profile in the multimode POF (patch) and SPR-POF sensor changed by shifting the SPR spectra. 

The force was changed in the developed setup by applying different weights to the patch between the holder’s clamps. In this way, the effective index (n_eff_) and the light propagation of the multimode waveguide (POF patch) feeding the SPR sensor varied as described in the outline reported in Figure 1b. In other words, the variation in force caused a change in incidence angles associated with each propagated mode in the SPR-POF sensor by changing the SPR condition, such as the resonance wavelength used in these measurements. Figure 1c shows the force in terms of weight applied at the POF patch. 

The SPR-POF sensor reported in Figure 1 is simple to realize via three steps, as extensively described in [25]. It was used with a fixed solution (water solution) over its gold sensing surface. In particular, this SPR-POF sensor was obtained by fixing the POF in a trench of resin support to realize the D-shaped POF sensing area by removing the cladding and part of the core via two different polishing papers. Next, a thin polymer layer was deposited on the D-shaped POF planar region by spinning and oven-curing steps. This polymer layer presented a refractive index major than the POF core to improve plasmonic performances [25]. Finally, in the last step, a nanofilm of gold (about 60 nm) was deposited by a sputter coater. An outline of this SPR-POF sensor is shown in Figure 1b, whereas its picture is reported in Figure 1c.

The experimental setup used to monitor the developed force sensor is shown in Figure 1c. In addition to the POF patch with the holder and the SPR-POF sensor elements, the setup was composed of two components: a white light source and a spectrometer. The white light source presented a spectral range extending from 360 nm to 1700 nm, whereas the spectrometer revealed a detection range between 350 nm and 1023 nm. Ocean Optics company (Dunedin, FL, USA) produces and sells these devices reported in Figure 1c (light source: model HL-2000-LL; spectrometer: model FLAME-S-VIS-NIR-ES).

In this work, the applied force variation on the POF patch was measured via the resonance wavelength change by keeping constant the refractive index of the bulk solution over the SPR-POF sensor. In particular, the SPR wavelength shifted on the left when the applied weight (the force) on the patch increased. Therefore, the sensor’s sensitivity (S) can be defined as the absolute shift in resonance wavelength per unit change of force:

(1) $$S\;\left(n_{s}\right)=\left|{\left(\frac{{\delta \lambda }_{\mathrm{SPR}}}{\delta F}\right)}_{n_{s}}\;\right|\left[\frac{\mathrm{nm}}{N}\;\right]$$

where δF is the variation of the applied force on the patch (realized via applied weights), giving rise to a resonance wavelength shift equal to ${\delta \lambda }_{\mathrm{SPR}}$ at a constant refractive index of the bulk solution (n_s_) on the SPR-POF sensor. Another sensor parameter used to describe the performances is the sensor’s resolution (ΔF), which can be defined as:

(2) $$\Delta F={\left(\frac{\delta F}{{\delta \lambda }_{\mathrm{SPR}}}\right)}_{n_{s}}\delta M=\frac{1}{S\left(n_{s}\right)}\;\delta M\;\left[N\right]$$

where δM is the max experimentally measured standard deviation of the resonance wavelength (equal to about 0.1 nm).

## 3. Experimental Results and Discussion 

To experimentally test the force sensor system, different weights were applied on the POF patch, fixed between the holder’s clamps, when a constant water solution with a refractive index equal to 1.332 RIU (n_s_) was present over the gold surface of the SPR-POF sensor. The experimental results were repeated five times to test the sensor’s reproducibility and to detect the max standard deviation of the resonance wavelength.

**Figure 2:**
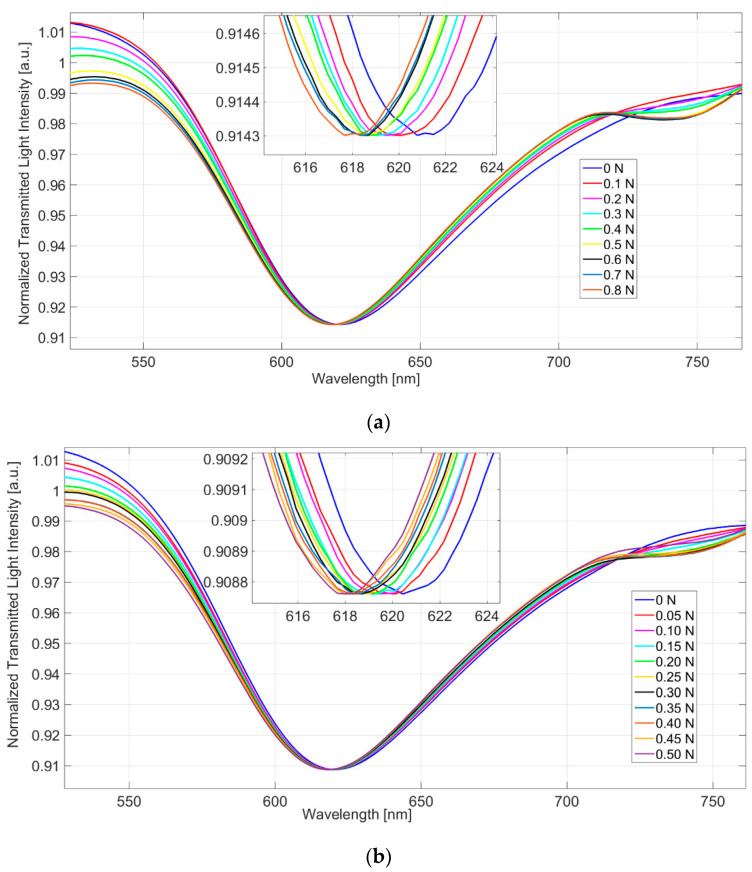
SPR spectra, experimentally obtained by normalization of the spectra acquired at different applied forces (when a fixed solution is present on the SPR-POF sensor) with the spectrum acquired without the force and when air is present on the SPR-POF sensor. (**a**) SPR spectra obtained by changing the steps of the applied force of 0.1 N in a range from 0 N to 0.8 N; (**b**) SPR spectra obtained by changing the applied force steps of 0.05 N in a range from 0 N to 0.5 N.

The experimental results demonstrated that the sensor’s reproducibility was good for an applied force ranging from 0 N to 0.8 N. More specifically, when the applied force (the weight) increased, the resonance wavelength decreased and shifted on the left (see Figure 2). Considering all the experimental tests, the max measured standard deviation of the resonance wavelength (δM) equaled about 0.1 nm.

Figure 2 shows the SPR spectra at different applied weights, obtained by a constant water solution (n_s_ = 1.332 RIU) present on the SPR-POF sensor. The SPR spectra reported in Figure 2 were obtained by normalization of the transmitted spectra at different applied forces with a reference spectrum (spectrum acquired when the SPR-POF sensor was in air and no force was applied on the patch). In more detail, Figure 2a shows the SPR spectra obtained by changing the steps of the applied force of 0.1 N, and adding weights of 10 g, in a range from 0 N to 0.8 N. Figure 2b shows the SPR spectra obtained by changing the applied force steps of 0.05 N, adding weights of 5 g in a range from 0 N to 0.5 N.

**Figure 3:**
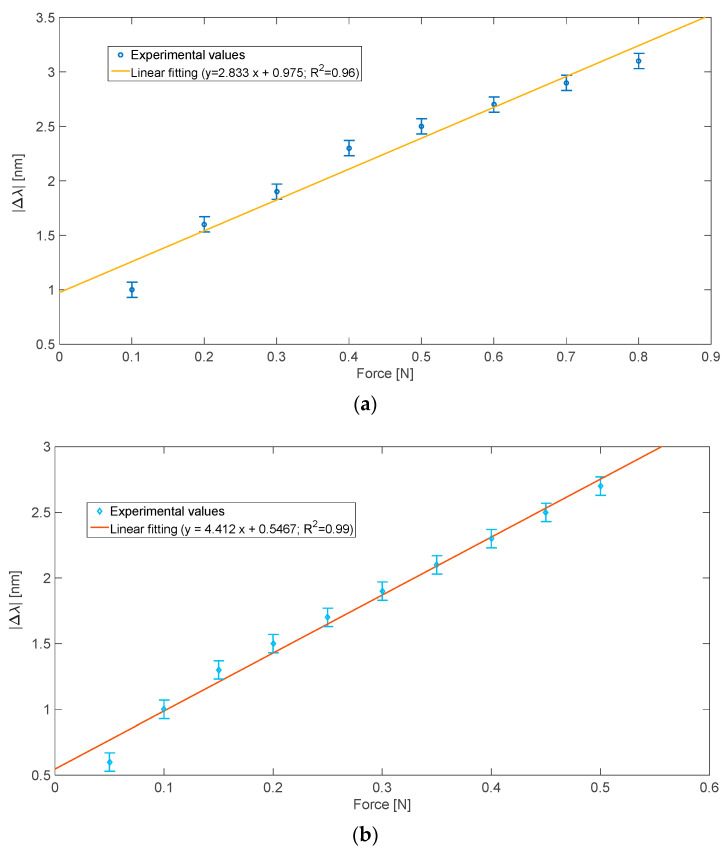
Absolute SPR wavelength variation (|Δλ|) versus applied force and linear fitting of the experimental data. (**a**) |Δλ| versus applied force obtained by changing the steps of the applied force of 0.1 N in a range from 0 N to 0.8 N. (**b**) |Δλ| versus applied force obtained by changing the applied force steps of 0.05 N in a range from 0 N to 0.5 N.

Figure 3 shows the absolute resonance wavelength variation (|Δλ|), calculated with respect to no applied force configuration, for both the results reported in Figure 2. More specifically, Figure 3 shows the results obtained by two different applied force variation steps relative to diverse force ranges, in a similar way to Figure 2.

Each experimental value reported in Figure 3 is the average of five different measurements, repeated in the same conditions. In this sensor system, in contrast with the disposable biosensor configurations; the same SPR-POF sensor can be used all times after the calibration curve. Figure 3 also reports the error bars corresponding to the highest measured standard deviation (equal to 0.1 nm) and the linear fittings of the experimental data.

From Equation (1), the force sensor sensitivity in the considered applied force range can be approximated with the slope of the linear fitting reported in Figure 3. Furthermore, the resolution (ΔF) of the proposed sensor can be obtained from these sensitivity values and Equation (2). In particular, considering the applied force range from 0 N to 0.5 N, where a better linear response was present in terms of Pearson’s correlation coefficient (R^2^ = 0.99), the sensor’s resolution was equal to about 22 mN.

Table 1 reports several examples of force sensors reported in the literature to compare the obtained sensors’ resolution with different sensing methods implemented via optical fibers [10,26,27,28]. 

As shown in the results reported in Table 1, the proposed force sensor presents performances comparable to the sensors based on the measurements of the intensity reflected signal [26] and FBG-based sensor [28]; moreover, it was worse than those based on whispering gallery mode resonators (PMMA-based) [10], and better than those obtained by exploiting sensors based on light intensity modulation due to POF bending [8,27].

## 4. Conclusions

We developed and analyzed a force sensor based on POFs. The proposed sensor was experimentally tested in different force ranges. The sensor demonstrated a good linear response in an applied force range from 0 N to 0.5 N, with a resolution value equal to 22 mN and a sensitivity value of about 4.4 nm/N. The proposed sensing approach opens the door to realize innovative POF sensors that, via special patches, change the resonance conditions in SPR multimode optical fiber sensors, with a force sensing area distant from the transducer (the SPR sensor) and the equipment. Moreover, by changing the kind of POF patch (e.g., diameter, material, etc.), the sensor’s performances can be changed in terms of sensitivity and applied force range.

## Figures and Tables

**Table 1:** Resolution comparison of force optical fiber sensors.

Sensor Configuration	Sensing Principle	Resolution	References
SPR-POF-based	Modification of the SPR conditions	~22 mN	This work
Reflective measurements with optical fibers	Measurements of the Intensity reflected signal	~10 mN	[26]
Whispering gallery mode resonators based	Whispering-gallery modes of dielectric resonators	~10 µN	[10]
Intensity-based POF beam	Modulation of light intensity via POFs macrobending	~0.1 N	[8,27]
Sensor System Based on Tri-Axial FBG with Flexure Structure	Fiber Bragg grating	~0.01 N	[28]

## Data Availability

The data are available on reasonable request from the corresponding author.
